# Avoiding extinction under nonlinear environmental change: models of evolutionary rescue with plasticity

**DOI:** 10.1098/rsbl.2021.0459

**Published:** 2021-12-08

**Authors:** Philip B. Greenspoon, Hamish G. Spencer

**Affiliations:** Department of Zoology, University of Otago, Dunedin 9016, New Zealand

**Keywords:** extinction, transgenerational plasticity, accelerating environmental change, model, adaptation, phenotypic plasticity

## Abstract

Rapid environmental changes are putting numerous species at risk of extinction. For migration-limited species, persistence depends on either phenotypic plasticity or evolutionary adaptation (evolutionary rescue). Current theory on evolutionary rescue typically assumes linear environmental change. Yet accelerating environmental change may pose a bigger threat. Here, we present a model of a species encountering an environment with accelerating or decelerating change, to which it can adapt through evolution or phenotypic plasticity (within-generational or transgenerational). We show that unless either form of plasticity is sufficiently strong or adaptive genetic variation is sufficiently plentiful, accelerating or decelerating environmental change increases extinction risk compared to linear environmental change for the same mean rate of environmental change.

## 1. Introduction

Human impacts are causing rapid environmental change via environmental degradation and climate change. Changing environments pose a threat to the persistence of species [1]. Some species will be able to avert extinction through migration to habitats with favourable environments. Others will only be able to survive if functional traits change by phenotypic plasticity or evolution [2].

Evolutionary rescue is said to occur when evolutionary adaptation causes a population to avoid extinction [3]. It is relevant to conservation biology, where the goal is to reduce the rate of extinction of endangered species, as well as epidemiology where the goal is to increase the extinction rate of pathogens [4]. Modelling has highlighted when evolutionary rescue is most likely. For example, it is more likely with greater additive genetic variation or heritability of the adaptive phenotype [5]. Experimental evolution has shown evolutionary rescue in action. These experiments have confirmed theoretical predictions, such as the positive dependence of the probability of evolutionary rescue on genetic variation [6]. Demonstrating evolutionary rescue in the wild is challenging because it requires both demographic and population genetic data over multiple generations [3,7], though suggestive examples exist (e.g. [8]).

Phenotypic plasticity is an alternative route through which species may avert extinction. While most theory on plasticity’s role in preventing extinction has focused on within-generational plasticity [2,9,10], transgenerational plasticity also has potential to help avert extinction [11]. Transgenerational plasticity occurs when environmental information received by a past generation (e.g. parents) informs the phenotypic response of an individual. It may occur as the result of parental transmission of factors such as antibodies or by epigenetic inheritance. Transgenerational plasticity is expected to be less beneficial than within-generation plasticity when parental environments and offspring environments are weakly correlated, causing information received by parents to predict poorly the optimum offspring phenotype [12,13].

Rather than changing at a constant, linear rate, the pace of environmental change has been accelerating by various measures [14–19]. Although much theoretical work has considered how species may adapt to avoid extinction in the face of linear environmental change [2,20], none has considered the response to accelerating (or decelerating) change. Models not accounting for nonlinearity in climate change may overestimate the ability of species to adapt (whether genetically or plastically) to avoid extinction. As we try to assess and predict the consequences of climate change, it is critical to understand how species respond to the variety of environmental trajectories that they may encounter. Here, we work towards this goal with a simple model of a species that can adapt through evolution, within-generation plasticity or transgenerational plasticity, and is faced with a gradually changing environment in which change is accelerating, decelerating or linear. We investigate conditions under which evolution and plasticity can help avert extinction for a range of trajectories of environmental change.

## 2. Model

Previous work investigated the roles of evolution and phenotypic plasticity in reducing maladaptation and averting extinction [2]. We build on this work in two ways: first by considering an environment whose change through time can accelerate or decelerate, and second by considering the role of transgenerational plasticity (TGP) in addition to within-generation plasticity (WGP). To maintain continuity with this previous work [2], we make use of, and adapt, its notation, equations and parameter values where appropriate.

We assume a population lives in an environment that changes through time. The environment, *ε*, at time *t* is

2.1 $$\epsilon =\eta t^{\alpha },$$

with *η* linearly scaling the average rate of change of the environment, and *α*, the shape parameter, indicating the acceleration (*α* > 1) or deceleration (*α* < 1) of the environmental change. The population has a generation time of length *T*, which serves as a conversion factor between time, *t*, and number of generations, *n*.

The relative benefits of within-generation versus transgenerational plasticity are expected to depend on how well the cue-detection environment correlates with the selective environment. In our deterministic model of environmental change, these correlations are strong, making differences between within-generation and transgenerational plasticity difficult to discern. To facilitate distinguishing the abilities of within-generation versus transgenerational plasticity to prevent extinction, we built an alternate version of our model, which includes stochasticity in the environment that weakens the correlation between the selective environment and the environment at detection time (described in electronic supplementary material, section S1).

The individuals of the population have a phenotype, *z*, the sum of a genetic component and a plastic component. It is under Gaussian stabilizing selection with the optimum dictated by the environment and strength of stabilizing selection $\gamma_{z}=1/(\omega_{z}^{2}+\sigma_{z}^{2})$, where $\omega_{z}^{2}$ is the width of the fitness function and $\sigma_{z}^{2}$ is the total genetic variation of the phenotype. The genetic component of the phenotype is assumed to be controlled by many loci of small effect (i.e. is a normally distributed quantitative trait) with additive genetic variation $\sigma_{A}^{2}=\sigma_{z}^{2}h^{2}$ where *h*^2^ is the heritability of the phenotype. The plastic component includes within-generation plasticity or transgenerational plasticity. For the sake of generality, transgenerational plasticity can go back any number of generations, although in our simulations we focus on the special case of parental effects.

Plasticity is modelled as a linear function of either the environment in the present as detected by the individual (within-generation plasticity) or the environment detected by the individual’s parents (transgenerational plasticity). The environment-detection time for generation *n* is *t*_*n*,*i*,det_ = *T*(*n* − *i* − *τ*_*i*_) where *i* = 0 for within-generation plasticity and *i* = 1 for transgenerational plasticity. The parameters *τ*_*i*_ allow the detection of cues for within-generation plasticity and transgenerational plasticity to occur at arbitrary times, not necessarily at the boundary between generation times. The environment at this time is $\epsilon_{n,i,\det}=\eta t_{n,\det}^{\alpha }$ and the individual’s phenotype is additively calculated to be

2.2 $$z_{n,i}=a+b_{i}\epsilon_{n,i,\det}+e,$$

where *a* is the reaction norm intercept (i.e. the genetic component of phenotype), *b*_*i*_ is the strength of within-generational plasticity (*i* = 0) or transgenerational plasticity (*i* = 1) and *e* is a residual, normally distributed effect on the phenotype with mean 0 and variance, $\sigma_{e}^{2}$. When *α* = 1 and *i* = 0, equation (2.2) becomes the equation used by [2]. Because our focus for simulation analysis is on within-generation or parental effects, there is no issue that more information becomes available to individuals as simulations progress. To ensure that within-generation and transgenerational plasticity operate the same number of generations, we do not implement plasticity in the first generation in our simulations.

We consider evolution in the trait, *a*, while plasticity, generation time and cue-detection timing are fixed. Taking the average of equation (2.2), the mean phenotype in the population in generation *n* is

2.3 $${\bar{z}}_{n,i}={\bar{a}}_{n}+b_{i}\epsilon_{n,i,\det},$$

and the change in the mean phenotype across a generation is

2.4 $$\Delta {\bar{z}}_{n,i}=\Delta \bar{a}+b_{i}(\epsilon_{n+1,i,\det}-\epsilon_{n,i,\det}).$$

The value of $\Delta \bar{a}$ describes the genetic evolution of the trait, which depends on the strength of stabilizing selection. At any time, there is an optimum phenotype, *θ* = *Bε*, which is determined by the current environment, where *B* is the sensitivity of the optimum to environmental change and *ε* is the current state of the environment. Assuming that there is stabilizing selection on the phenotype, the change in reaction norm intercept is

2.5 $$\Delta \bar{a}=-\gamma_{z}(\bar{z}-\theta )\sigma_{A}^{2},$$

where *γ*_*z*_ is the strength of stabilizing selection on the phenotype and $\sigma_{A}^{2}$ is the additive genetic variance in *a* [2].

Change in population size is concurrent with genetic change, and adaptation to the environment results in positive population growth. The reproductive rate of a population with mean phenotype $\bar{z}$ is

2.6 $$r=r_{\max}-\frac{\gamma_{z}{\left(\theta -\bar{z}\right)}^{2}}{2T}.$$

where *r*_max_ is the growth rate for a perfectly adapted population. The change in population size is then calculated as

2.7 $$\Delta N=\left\{ \begin{array}{ll} rN(1-N/K)\; & \mathrm{when}\;r>0\;\\ rN\; & \mathrm{when}\;r\leq 0.\; \end{array}\right.,$$

where *K* is the carrying capacity of the population. Logistic population growth is assumed when *r* > 0 to keep the population size at or below the carrying capacity, while exponential decay is assumed when *r* < 0 to ensure that a population at the carrying capacity still declines.

**Figure 1.**
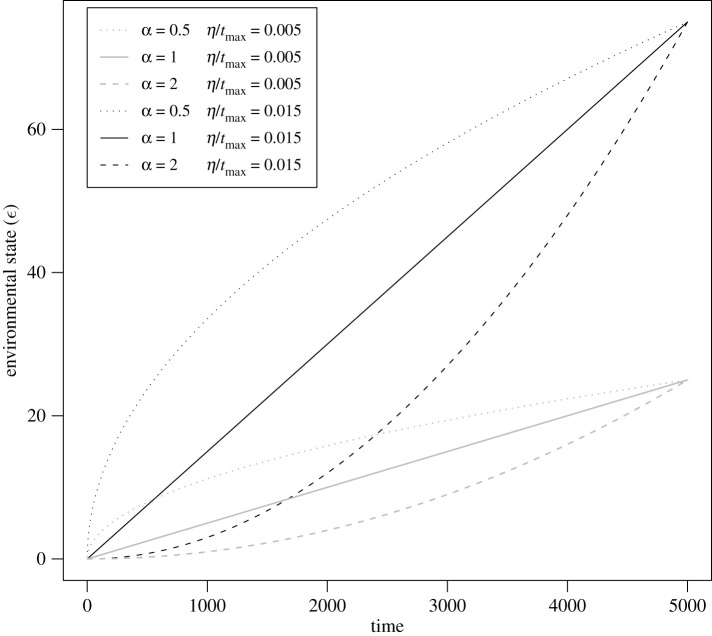
Sample trajectories of decelerating, linear, and accelerating environmental change for two different values of average environmental change, $\frac{\eta }{t_{\max}}$.

In order to compare results across exponents, *α*, which describe the degree to which the environmental change is accelerating (*α* > 1) or decelerating (*α* < 1), we fix the total amount of environmental change that occurs across the range of values of *α* for a given value of *η* (see equation (2.1)). Hence, when we compare outcomes at the end of simulations, the total amount of environmental change that has occurred is the same for all values of *α*, and all that differs is the environment’s trajectory, whether linear, accelerating or decelerating. In other words, the mean rate of environmental change per generation is the same. We control for total amount of environmental change by replacing *t* in equation (2.1) with $\frac{t}{t_{\max}}$, so that the equation becomes *ε* = *η*(*t*/*t*_max_)^*α*^, where *t*_max_ is the total simulation runtime, which is the same across all simulations. Likewise, the detection time becomes $t_{n,i,\det}=\frac{T(n-i-\tau_{i})}{t_{\max}}$. Examples of resulting environmental trajectories are shown in figure 1 with two different values for *η* and exponents, *α*, representing decelerating change (*α* < 1), linear change (*α* = 1) and accelerating change (*α* > 1).

By controlling for the average rate of environmental change, we unavoidably cause environmental trajectories to differ in their maximum rates of environmental change. Potentially, variation in the maximum rates of environmental change could cause variation in extinction rates. For example, we would intuitively expect decelerating environmental change to cause less extinction than linear or accelerating change. However, by controlling for the average rate of environmental change, decelerating trajectories are constrained to include an early rapid change in the environment, which could cause extinction to happen near the beginning of the simulation. To address this issue, we built an alternative version of our model in which we control for the maximum rate of environmental change rather than average rate of environmental change (described in electronic supplementary material, section S2).

## 3. Results

**Figure 2.**
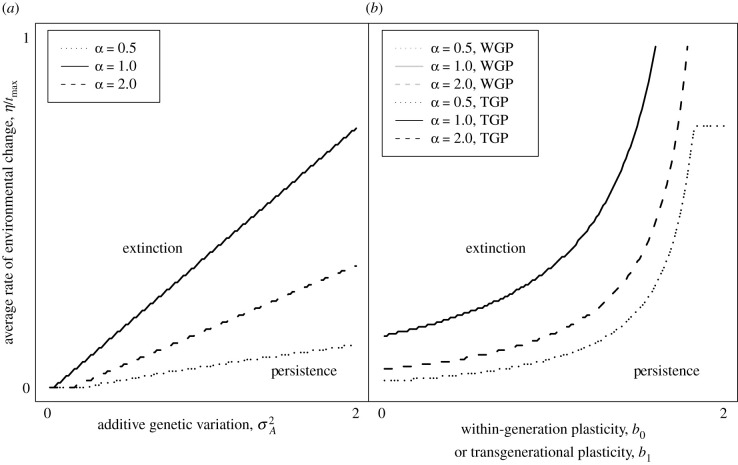
Extinction versus persistence of a population depends on the level of additive genetic variation ($\sigma_{A}^{2}$, panel *a*) or plasticity (*b*_*i*_, panel *b*) and the shape of the environmental change, *α*. The boundary between extinction and persistence is shown for decelerating (*α* = 0.5), linear (*α* = 1) and accelerating (*α* = 2) environmental change. Other parameter values are: *B* = 2, *T* = 1, *ω*_*z*_ = 49, *h*^2^ = 0.5, *t*_max_ = 5000, *r*_max_ = 0.14, *K* = 10^5^, *τ*_0_ = *τ*_1_ = 0.1.

At the end of each simulation, we record whether or not the population went extinct, where extinction is defined as either the population size *N* having declined below 1 or the value of *r* being less than 0 at the end of the simulation, implying an extinction debt. Without phenotypic plasticity (*b*_*i*_ = 0 for all *i*), the population’s ability to persist depends on genetic evolution (i.e. evolution of the intercept, *a*; equation (2.5)). Populations with more additive genetic variation $\sigma_{A}^{2}$ are therefore able to withstand greater average rates of environmental change, *η*/*t*_max_ (figure 2*a*, which shows regions of parameter space where persistence is predicted and regions where extinction is predicted). With more additive genetic variation, populations are better able to persist despite acceleration or deceleration of the environmental change, *α*, reflected in a larger area of the region of parameter space where persistence is predicted (figure 2*a*). Note that any horizontal slice through parameter space holds the average rate of environmental change constant, allowing for controlled comparisons of extinction risk among values of *α*. The boundaries between parameter space regions of extinction and persistence are linear: an increase in additive genetic variation produces a proportionate increase in tolerance to environmental change.

Except for the extreme form of plasticity that achieves a nearly perfectly adaptive phenotype every generation, extinction avoidance requires genetic evolution in our models: therefore, we do not consider models with only plasticity and no genetic variation. In models including plasticity and genetic variation, within-generation plasticity (*b*_0_ > 0 and *b*_*i*_ = 0 for all *i* ≠ 0) and transgenerational plasticity (*b*_1_ > 0 and *b*_*i*_ = 0 for all *i* ≠ 1) each increase the population’s tolerance of environmental change (figure 2*b*), and are indistinguishable in their effects. Although within-generation plasticity achieves a slightly superior adaptive plastic response, populations with transgenerational plasticity compensate for their inferior adaptive plastic response with better adaptation through genetic change in the phenotype (not shown).

While the shapes of the boundaries in parameter space between regions of persistence and regions of extinction are similar to those found by [2] in their investigation of linear environmental change (*α* = 1), the areas of the regions change with acceleration/deceleration in the environmental change, *α*: with either decelerating change, *α* < 1, or accelerating change, *α* > 1, the population is less tolerant to environmental change. The boundaries between regions of extinction and persistence are concave up: an incremental increase in plasticity produces a greater increase in tolerance to environmental change when plasticity is already high than when plasticity is at a low level. The slopes (not shown) of the boundary-separating curves differ among values of *α* for both genetic variation (figure 2*a*) and plasticity (figure 2*b*). Therefore, genetic variation and plasticity each interact with *α* to determine a population’s tolerance to environmental change. However, it appears that this interaction is stronger for genetic variation (i.e. the slopes differ more for the different values of alpha), suggesting that genetic variation’s ability to prevent extinction depends more on the shape of the environmental change, *α*, than plasticity’s.

Extinction times for populations experiencing accelerating environmental change tend to be later than for populations experiencing decelerating environmental change (electronic supplementary material, figure S1). Even though we control for the average rate of environmental change in our environmental trajectories, when it is decelerating, the fastest change happens early, while when there is acceleration the fastest change happens late. These bursts of change explain why nonlinear environmental trajectories are more challenging to populations than linear trajectories and more likely to result in extinction. When the fastest change happens early rather than late (i.e. comparing decelerating to accelerating change), the population is more vulnerable to extinction (the larger extinction region for declerating change in figure 2). While constraining average rates of change to be equal, it is unavoidable that environmental trajectories differ in their maximum rates of environmental change, resulting in the counterintuitive result that decelerating environmental change is the most conducive to extinction. However, controlling for the maximum rate of environmental change rather than the mean rate of environmental change causes populations experiencing decelerating environmental change to be the least susceptible to extinction (electronic supplementary material, figure S2).

The finding of nearly identical extinction risks between within-generation and transgenerational plasticity is due to the cue information being a highly reliable indicator of selective environment in both cases (i.e. the correlation between detection and selective environments is strong). In our stochastic model, the environmental stochasticity disrupts the reliability of transgenerational cues more than within-generational cues, allowing differences to be revealed. Within-generational plasticity becomes noticeably superior to transgenerational plasticity in its ability to avert extinction (electronic supplementary material, figure S3).

## 4. Discussion

For species unable to migrate, adaptation through evolution and/or phenotypic plasticity may be the only means to prevent their extinction [2], setting up a race between environmental change and adaptation. This issue is particularly urgent in locations with significant human-disturbance, as habitat fragmentation may bar species from migrating to habitats with more favourable environmental conditions [21].

A large body of theory has addressed how the probability of evolutionary change being sufficient to stave off extinction (i.e. evolutionary rescue) depends on features of both the population and the environmental change [3]. For example, the probability increases with the level of standing genetic variation, and decreases with generation time of the population and as the rate of environmental change increases. Missing from this body of theory has been consideration of the functional forms of the change in environmental variables, and, particularly pertinent, whether it is accelerating or decelerating. Acceleration in environmental change could be directly caused by acceleration in human activities such as emissions, or by synergisms among changes in multiple environmental factors [22]. For example, global warming, while being itself a stress to species, also can increase the risk of disease [23], such as was the case with amphibians infected by chytrid fungus [24].

We show that either acceleration or deceleration of environmental change reduces a population’s ability to withstand environmental change. It is important to note that in our comparisons, we control for the total amount of environmental change (and thus the mean per generation rate of environmental change), and all that differs is the trajectory of the environmental change through time (figure 1). Genetic evolution (figure 2*a*) and/or phenotypic plasticity (within generation or transgenerational; figure 2*b*) can promote persistence despite accelerating or decelerating environmental change. For simplicity, we assumed that plasticity and genetic variation are fixed rather than evolving. An individual-based model, which relaxed both of these assumptions, showed that within-generation plasticity can evolve in the face of a changing environment and thereby help the population avoid extinction [25]. However, if costly, the evolved plasticity could instead promote extinction. Including evolving plasticity, both within-generation and transgenerational, as well as costs of plasticity, into a model with nonlinear environmental trajectories would be a fruitful avenue for future work.

With deterministic environmental change, there is little difference between within-generation plasticity and transgenerational plasticity in the adaptive phenotypic response that is achieved. Even though within-generation plasticity achieves a slightly better fit to the environment through plasticity, populations exhibiting transgenerational plasticity compensate by achieving slightly superior genetic adaptation. However, with stochasticity in the environment, which disrupts the correlation between the selective environment and the detected environment more for transgenerational plasticity than within-generation plasticity, within-generation plasticity becomes qualitatively superior to transgenerational plasticity in helping to avert extinction (electronic supplementary material, figure S3).

The study of evolutionary rescue can inform species conservation efforts. In addition to helping to predict which species have the right conditions for evolutionary rescue, the principles of evolutionary rescue can be applied by conservation biologists and managers to increase the chances of species survival. Careful assisted migration or gene flow within a species’ range has the potential to reduce maladaptation of a species [26,27]. Browne *et al.* [28] recently demonstrated the potential of genome-informed assisted gene flow to avert maladaptation due to climate change. They identified individual trees as being more or less genomically equipped to cope with warmer climates, and predicted that using those trees as seed sources would help avert population declines in the face of warming conditions. When making decisions like this, it would also be useful, though logistically more challenging, to select individuals for the strength of their phenotypic plasticity [29] (represented by parameters *b*_0_ for within-generation plasticity and *b*_1_ for transgenerational plasticity). Our results suggest that if the environmental change is accelerating, it is even more critical to ensure that individuals have either strong phenotypic plasticity or high levels of adaptively relevant genetic variation than when the environmental change is linear.

## Data accessibility

This is a theoretical study, and simulations of our models produced the data shown in the figures. The simulation code and data are available from the Dryad Digital Repository: https://doi.org/10.5061/dryad.prr4xgxnf [30]. A description of our dataset is contained within this Dryad depository in the file ‘description_of_data.pdf’.
